# Beneficial Effects of Green Tea Catechins on Female Reproductive Disorders: A Review

**DOI:** 10.3390/molecules26092675

**Published:** 2021-05-03

**Authors:** Datu Agasi Mohd Kamal, Norizam Salamt, Siti Sarah Mohamad Zaid, Mohd Helmy Mokhtar

**Affiliations:** 1 Department of Physiology, Faculty of Medicine, Universiti Kebangsaan Malaysia, Kuala Lumpur 56000, Malaysia; datuagasi90@gmail.com (D.A.M.K.); norizam_salamt@ukm.edu.my (N.S.); 2 Department of Biomedical Sciences and Therapeutics, Faculty of Medicine and Health Sciences, Universiti Malaysia Sabah, Kota Kinabalu 88400, Malaysia; 3 Department of Environment, Faculty of Forestry and Environment, Universiti Putra Malaysia, Serdang 43400, Malaysia; mz_sarah@upm.edu.my

**Keywords:** catechin, epigallocatechin-3-gallate, polycystic ovary syndrome, endometriosis, dysmenorrhea

## Abstract

Tea is one of the most widely consumed beverages worldwide after water, and green tea accounts for 20% of the total tea consumption. The health benefits of green tea are attributed to its natural antioxidants, namely, catechins, which are phenolic compounds with diverse beneficial effects on human health. The beneficial effects of green tea and its major bioactive component, (−)-epigallocatechin-3-gallate (EGCG), on health include high antioxidative, osteoprotective, neuroprotective, anti-cancer, anti-hyperlipidemia and anti-diabetic effects. However, the review of green tea’s benefits on female reproductive disorders, including polycystic ovary syndrome (PCOS), endometriosis and dysmenorrhea, remains scarce. Thus, this review summarises current knowledge on the beneficial effects of green tea catechins on selected female reproductive disorders. Green tea or its derivative, EGCG, improves endometriosis mainly through anti-angiogenic, anti-fibrotic, anti-proliferative and proapoptotic mechanisms. Moreover, green tea enhances ovulation and reduces cyst formation in PCOS while improving generalised hyperalgesia, and reduces plasma corticosterone levels and uterine contractility in dysmenorrhea. However, information on clinical trials is inadequate for translating excellent findings on green tea benefits in animal endometriosis models. Thus, future clinical intervention studies are needed to provide clear evidence of the green tea benefits with regard to these diseases.

## 1. Introduction

Tea is originated from East Asia and has become the most widely consumed beverage worldwide after water [1]. Green tea, black tea and oolong tea are harvested from the same *Camellia sinensis* plant but possess different properties (Table 1). Green tea accounts for 20% of the total tea consumption worldwide and is the primary beverage consumed daily in Asian countries [2], including Japan [3], China [4] and Korea [5]. Green tea is consumed in an average of three cups and as high as 10 cups per day [6]. According to a human adverse event data, the observed safe level for green tea is 704 mg of epigallocatechin-3-gallate (ECGC)/day, which is approximately 880 mL of brewed green tea daily [7].

Freshly plucked tea leaves are steamed or pan-fried immediately during green tea production. This step is essential to the inactivation of polyphenol oxidase and native microflora that eventually inhibit the aerobic oxidation of tea catechins during tea fermentation [14]. Prolonged fermentation process decreases the antioxidant capacity and the levels of monomeric catechins and simple phenolic acids [14]. Thus, a non-fermentation process in green tea production contributes to its green colour (hence the name), high polyphenol content and better radical-quenching ability compared with that of black tea [15,16].

Phenolic compounds of green tea, such as catechins, act as natural antioxidants and constitute 6–16% of its dry leaves [17]. The four major green tea catechins include (-)-epigallocatechin-3-gallate (EGCG), (-)-epigallocatechin (EGC), (-)-epicatechin-3-gallate (ECG) and (-)-epicatechin (EC) [18]. Catechins possess excellent antioxidant properties and superior to glutathione, vitamin C and flavonoids [19]. These catechins neutralise free radicals and facilitate the detoxification of enzymes, such as catalase, glutathione peroxidase and glutathione reductase [20,21]. Numerous studies showed the various beneficial effects of green tea extract and its catechins on health, including high antioxidant [22], osteoprotective [23], neuroprotective [24], anti-cancer [25,26], anti-hyperlipidaemia [27] and anti-diabetic [28] effects, and that green tea extract and its catechins improve fertility in humans and animals [18].

The daily consumption of green tea has positive effects on the male and female reproductive systems. A meta-analysis study demonstrated that at least seven cups of green tea per day could effectively prevent prostate cancer [25]. Studies in lead-induced rats treated with green tea extracts showed improved male fertility, as indicated by significant increases in sperm count, motility and testosterone level relative to those in the control group. This effect may be due to the ability of green tea to inhibit the absorption and promote the excretion of heavy metals [29]. Meanwhile, the effect of green tea on the female reproductive system is demonstrated by a decrease in ovarian cancer risk among southern Chinese women who regularly drink green tea [30]. This finding is further supported by the shrinkage of total fibroid volume and reduced fibroid-specific symptom severity in women with symptomatic fibroids after 4 months of supplementation with 800 mg of green tea extract [31]. A high content of EGCG inhibits the proliferation of leiomyoma tumour (fibroid tumour) and induce apoptosis [32].

Green tea is a popular beverage with a wide range of ready-to-drink packs and supplements in the forms of tablets and capsules. Information on its health benefits, especially on the reproductive system, offers prospects of natural complementary treatments. However, the review of green tea’s health benefits on female reproductive disorders, including polycystic ovary syndrome (PCOS), endometriosis and dysmenorrhea, remains scarce. Therefore, this review aims to analyse studies related to the beneficial effects of green tea catechins on selected female reproductive disorders.

## 2. Methodology

A literature search was performed to identify and map out relevant and pertinent articles related to the beneficial effects of green tea catechins on female reproductive disorders. Peer-reviewed and full-text English articles were collected in a time frame from as early as 1960 to January 2021 from electronic databases, including Scopus, MEDLINE via EBSCOhost and Google Scholar. The following set of keywords was used: (1) Green tea or catechin and (2) PCOS or Endometriosis or Dysmenorrhea. The literature search was further supplemented by referencing related review articles and scientific reports found from the search results.

## 3. Beneficial Effects of Green Tea Catechins on Female Reproductive Disorders

**Figure 1:**
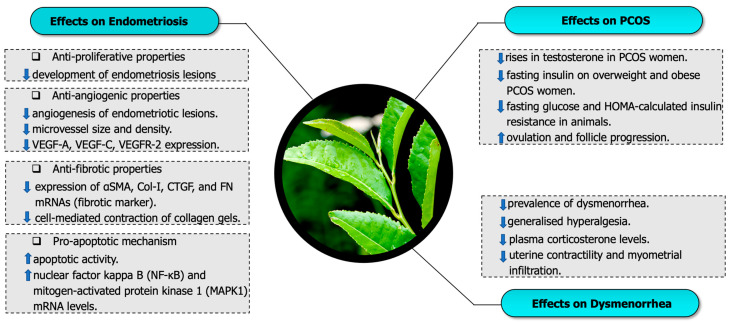
Beneficial effects of green tea catechins on endometriosis, polycystic ovary syndrome (PCOS) and dysmenorrhea.

In this section, the beneficial effects of green tea or its derivatives epigallocatechin-3-gallate are discussed according to their effects on selected female reproductive disorders. A total of 13 articles were discussed, comprising seven articles related to endometriosis, four articles associated with PCOS and two articles about dysmenorrhea. The beneficial effects of green tea or its derivatives, such as EGCG, were discussed, including effects against (a) endometriosis (b) PCOS and (c) dysmenorrhea (Figure 1).

### 3.1. Effects of Green Tea Catechins on Endometriosis

Endometriosis is a chronic disorder characterised by the implantation of endometrial glands and stroma outside the uterine cavity [33]. It affects adolescents and reproductive-aged women and is commonly associated with chronic pelvic pain and infertility [34]. EGCG, the most abundant component found in green tea, has a beneficial effect against endometriosis.

**Figure 2:**
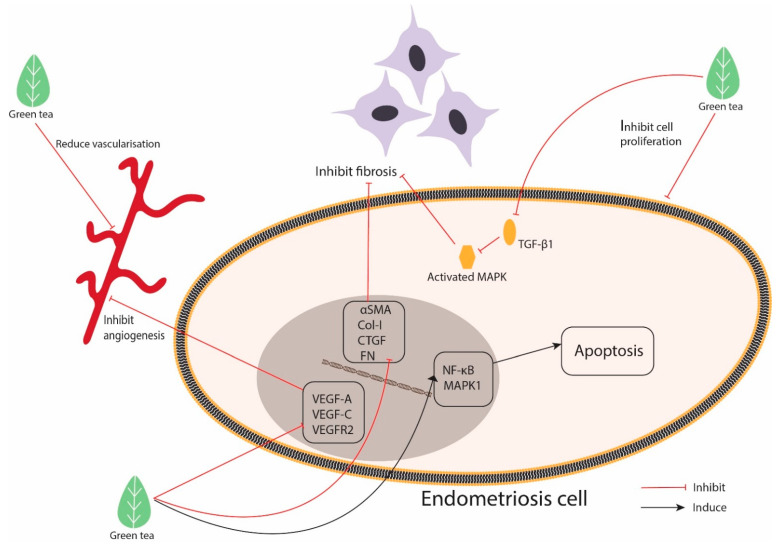
Mechanism related to the beneficial effects of green tea catechins on endometriosis.

In this review, we found seven studies exploring the effect of EGCG against endometriosis (Table 2: in vivo studies, and Table 3: in vitro studies). These studies utilised animal models of endometriosis, which involved an endometrium transplanted into an animal; the endometrium tissues were derived either from an endometriosis patient or from an animal endometrium. The studies reported the positive effects of EGCG on the endometrium, mainly those exerted by anti-angiogenic, anti-fibrotic, anti-proliferative and proapoptotic mechanisms. The anti-proliferative effect of EGCG against endometriosis was reported in most of the studies. EGCG causes regression in the development of endometriosis lesions assessed by size, number, volume, weight and cell proliferation [33,35,36,37,38]. Figure 2 illustrates a simplified version of the mechanism related to the beneficial effects of green tea catechins on endometriosis.

The anti-angiogenic properties of EGCG were reported in five studies [33,35,36,37,39]. Xu et al. [33] transplanted eutopic endometria from stage III endometriosis patients into severely compromised immunodeficient (SCID) mice. In this study, the mice are treated either with 20 mg/kg/day vitamin E as antioxidant control, 50 mg/kg EGCG or saline intraperitoneally for 3 weeks. Treatment with EGCG but not vitamin E reduced the level of angiogenesis in endometriotic lesions and its adjacent tissues, as indicated by the weak staining of CD34 and cytokeratin; they also reported decreases in microvessel size and density and down-regulation of mRNA for angiogenic vascular endothelial growth factor A (VEGF-A), further demonstrating the anti-angiogenic effect of EGCG. However, no changes were observed in hypoxia-inducible factor 1, alpha subunit (HIF1A) expression, suggesting that green tea EGCG has an anti-angiogenic effect in endometriosis, specifically through VEGF suppression [33]. Another study utilised both tissues from human with stage III endometriosis and mouse endometrial tissues transplanted into SCID mice. In this study, treatment with EGCG at 50 mg/kg through the intraperitoneal route again showing inhibition of angiogenesis mainly through the reduction of microvessel formation and down-regulation of vascular endothelial growth factor C (VEGF-C) and tyrosine kinase receptor VEGF receptor 2 (VEGFR2) expression [39]. In the in vitro part of this study, 10–50 µM EGCG inhibited VEGF-C/VEGFR-2 signalling through c-JUN, interferon-g, matrix metalloproteinase-9 and chemokine (C-X-C motif) ligand, which are the three pathways for endothelial proliferation, inflammatory response and mobility [39].

The anti-angiogenic properties of EGCG were demonstrated in another study utilising in vivo and in vitro methods [37]. In this study, endometrium and ovarian follicles transplanted into dorsal skinfold chamber models of Syrian golden hamsters. Treatment with 65 mg/kg EGCG selectively inhibited angiogenesis and the blood perfusion of endometriotic lesions without affecting blood vessel development in the ovarian follicles. These are indicated by a significantly reduced number and density of microvessels development and decreased centerline red blood cell velocity and volumetric blood flow within the endometriotic lesions but not in ovarian follicles. In the in vitro part of this study, 40 µM EGCG suppressed estrogen-stimulated activation, proliferation and VEGF expression of endometrial cells in isolated hamster endometrial cells [37]. Green tea EGCG has poor bioavailability [40]. Hence, a study utilised the prodrug of EGCG (EGCG octaacetate) to enhance the stability and bioavailability of EGCG. In this study, EGCG octaacetate resulted in a significantly lower degree of staining of CD31 and αSMA (angiogenic marker) compared with the EGCG group [36].

EGCG exerts anti-fibrotic effects against endometriosis in in vivo and in vitro settings [41]. In this study, the endometriosis model of mice treated with EGCG at 50 mg/kg/day for 14 days showed inhibited fibrosis, which was indicated by the weak Sirius red and Masson trichrome staining. Endometrial and endometriotic stromal cells cultured and treated with 50 or 100 µM EGCG showed inhibited cell proliferation, migration and invasion. EGCG suppressed the expression of αSMA, Col-I, CTGF and FN mRNAs (fibrotic marker) in endometriotic and endometrial stromal cells and attenuated the cell-mediated contraction of collagen gels at 8, 12 and 24 h. Moreover, EGCG inhibited the TGF-β1-stimulated activation of MAPK, and Smad signalling pathways further elucidated the mechanism of the anti-fibrotic effect of EGCG against endometriosis [41].

The proapoptotic feature of EGCG has been demonstrated in three studies which reported an increase in apoptotic activity in endometriosis cells after EGCG treatment [33,35,36]. Xu et al. [33] assessed the apoptotic activity of endometriosis in an endometriosis mouse model upon 50 mg/kg EGCG treatment through terminal deoxynucleotidyl transferase dUTP nick end labelling (TUNEL) assay and discovered apoptosis in the lesions was more prominent compared with that in the control group. They found that the nuclear factor kappa B (NF-κB) and mitogen-activated protein kinase 1 (MAPK1) mRNA levels (apoptosis marker) were elevated after EGCG treatment [33]. Endometriosis animal and in vitro studies that used TUNEL assays showed an increase in the level of apoptosis after treatment with 20 and 100 mg/kg EGCG orally and 40, 80 and 100 µM for the primary cultures of human endometrial epithelial cells of patients with endometriosis. Another study used the prodrug of EGCG (EGCG octaacetate) and found through TUNEL assay, that the number of apoptotic cells in the pro-EGCG group was significantly higher than that in the EGCG group [36].

EGCG exerts a beneficial effect against endometriosis. However, no study involving humans has explored the effect of green tea or EGCG on endometriosis. Further investigation may explore the relation of green tea or EGCG in patients with endometriosis to discover the beneficial effects of EGCG on humans.

### 3.2. Effects of Green Tea Catechins on PCOS

PCOS is a combination of complex reproductive, endocrine and metabolic syndrome [42]. Approximately 6–10% (defined by 1990 NIH criteria) of reproductive women have been affected by PCOS, and the incidence rates doubled according to Rotterdam or Androgen Excess-PCOS Society criteria [43]. Currently, PCOS has no definite treatment, symptomatic treatment alone is being implemented [44]. Green tea and its derivatives, catechins, have beneficial health effects against PCOS. We found three clinical trials and one animal study exploring the effect of green tea or its derivative catechin against PCOS (Table 4). Most of these studies showed the positive health effects against PCOS, suggesting that green tea is a supplement for currently available drugs for PCOS.

One of the factors that may increase the risk of developing PCOS in women is obesity [45]. Even 5–10% weight loss alleviates many PCOS features [42]. A clinical trial involving overweight and obese PCOS women who consumed green tea tablets (unspecified dose) showed a significant reduction in body weight compared with women in the placebo group [46]. Reduction in body weight was observed in PCOS-induced rats after treatment with hydro-alcoholic green tea extract through intraperitoneal injection [47]. However, no change in BMI and body weight was recorded in two clinical trials involving green tea tablets and PCOS women [48,49].

Chronic low-grade inflammation is a pathogenesis of PCOS [50]. Despite that green tea has anti-inflammatory properties [51], a clinical trial involving PCOS women who consumed 500 mg of green tea tablet, reported no significant difference among the levels of inflammatory marker TNF-α, IL-6 and hs-CRP [48]. Therefore, more studies should be carried out to prove the anti-inflammatory effect of green tea against PCOS.

The National Institutes of Health and Androgen Excess and PCOS Society consider hyperandrogenism one of the must-have criteria for diagnosing PCOS [45]. Green tea or its derivatives inhibits the increase in testosterone level in PCOS women [46] or PCOS-induced animals [47]. However, Chan et al. did not find any difference in testosterone, SHBG, free androgen, androstenedione, DHEA-S, FSH and LH levels between the green tea treatment group and placebo group in obese PCOS women [49].

Nearly half of women diagnosed with PCOS have insulin-resistant hyperinsulinism [52]. Green tea or its derivatives normalise hyperinsulinism in PCOS. A clinical trial recorded a significant reduction in fasting insulin on overweight and obese PCOS women after green tea tablet treatment [46]. However, Chan et al. found no change in fasting insulin, fasting glucose, 2 h post-load glucose and fasting G:I ratio in obese PCOS women who consumed capsules containing 2% freeze-dried tea powder for 3 months [49]. In the animal PCOS model, the study on the rat model found no difference in fasting insulin level, but significant reductions in fasting glucose and HOMA-calculated insulin resistance were recorded [47].

PCOS is the leading cause of anovulatory infertility in women [42]. In the PCOS animal model, green tea treatment improved ovulation and follicle progression and inhibited cyst formation. This result was assessed on the basis of the increment in the number of follicles and corpus luteum and reduced number of cystic follicles [47].

This section summarises and critically analyses the effects of green tea and catechins against PCOS. Many discrepancies were recorded among clinical trials and animal studies with regard to these effects. More studies are needed to explore the conclusive effect of green tea on PCOS. Studies discovering the mechanism of green tea effect against PCOS remains limited. Overall, green tea has beneficial effects against PCOS.

### 3.3. Effects of Green Tea Catechins on Dysmenorrhea

Dysmenorrhea refers as the occurrence of painful cramps of uterine origin during menstruation [53]. It has two types: primary dysmenorrhea, which refers to pain without any evidence of pathology [54], and secondary dysmenorrhea, which is caused by specific pelvic pathological conditions, such as adenomyosis, fibroids and endometriosis [55]. The prevalence of dysmenorrhea in reproductive age women ranges from 16.8% to 81% [56].

Green tea and EGCG have effects against dysmenorrhea (Table 5). A cross-sectional study involving 1183 reproductive-age women in Shanghai, China, found that the consumption of green tea is associated with a low prevalence of dysmenorrhea. Furthermore, this study reported a stronger relationship of green tea intake in moderate-to-severe dysmenorrhea than in mild dysmenorrhea. However, the exact amount and duration for green tea intake are not discovered in this study. This study examined the relation of consumption of caffeinated beverages (coffee) with dysmenorrhea to explore whether caffeine in tea was responsible for the relief of dysmenorrhea. The results showed that coffee was positively related to the severity of dysmenorrhea, suggesting that caffeine in tea might not be responsible for the effect against dysmenorrhea [57].

Another study treated mice with 1 mg/kg tamoxifen to induce adenomyosis [58]. The result showed that the induction of adenomyosis resulted in progressive generalised hyperalgesia. The amplitude and frequency of uterine contractions were elevated, contributing to dysmenorrhea. Elevated plasma corticosterone levels indicated stress level. Treatment with 5 and 50 mg of EGCG alleviated generalised hyperalgesia and reduced plasma corticosterone levels. In addition, EGCG reduced uterine contractility and suppressed myometrial infiltration. These results suggested that EGCG have effects against dysmenorrhea [58].

The cross-sectional study discussed above provided the relation between green tea intake and lowered prevalence of dysmenorrhea, whereas the animal study showed that the EGCG compound found in green tea alleviates dysmenorrhea. However, no mechanistic study has explored the possible molecular pathway of green tea’s beneficial effect against dysmenorrhea.

## 4. Conclusions

Green tea or its derivative EGCG possesses health benefits and effects against endometriosis, PCOS and dysmenorrhea according to human and animal studies. However, no clinical trial has been conducted to translate the excellent findings of green tea benefits in the animal endometriosis model. Future studies may focus on green tea benefit on PCOS and dysmenorrhea to elucidate the discrepancy recorded among current studies.

## Figures and Tables

**Table 1:** Different properties of green tea, oolong tea and black tea.

Properties			
	Green Tea	Oolong Tea	Black Tea
Consumption [8]	20%	<2%	75%
Main consumer [8]	Asian	Asian	European and North Americans
Preparation [9]	Non-fermented	Semi-fermented	Fully fermented
Characteristics [9]	Green colourRetain almost all original polyphenol contents	Combine freshness of green tea and the fragrance of black tea	Reddish black colour, mellow flavour, low bitterness and astringency
Chemical Profile			
Caffeine (mg/g) [10]	34.86 ± 4.32	19.67 ± 2.95	28.54 ± 3.68
Total catechins (mg/g) [11]	105.85 ± 35.69	86.91 ± 23.54	10.18 ± 6.68
Epigallocatechin-3-gallate (EGCG) (mg/g) [10,11,12]	18.10–54.06	7.36–38.36	2.19–9.18
Epigallocatechin (EGC) (mg/g) [10,11,12]	13.44–36.53	3.57–30.61	0.31–3.23
Epicatechin (EC) (mg/g) [10,11,12]	4.90–7.27	1.75–7.00	0.71–2.19
Epicatechin-3-gallate (ECG) (mg/g) [10,12]	5.34–17.10	3.07–5.09	2.65–8.92
Total Theaflavins (mg/g) [12]	0.88–5.56	0.66–3.63	10.70–17.28
L-theanine (mg/g) [11,13]	2.16–4.03	0.41–1.29	0.88–1.37

**Table 2:** In vivo studies found related to the effects of green tea catechins on endometriosis.

Treatment	Treatment Duration	Type of Study	Findings	Conclusion	References
1. 50 mg/kg/day green tea Epigallocatechin-3-gallate (EGCG) 2. 20 mg/kg/day vitamin E 3. Saline via intraperitoneal injection	Daily for 2 weeks.	Hetero-transplants of eutopic endometrium from patients with stage III endometriosis into severely compromised immunodeficient (SCID) mice	In the EGCG treated group: Endometriotic lesions were smaller than control. The glandular epithelium was smaller and eccentrically distributed. Angiogenesis in lesions and adjacent tissues was under-developed, and microvessel size and density were smaller than the control. mRNA for angiogenic vascular endothelial growth factor-A was significantly down-regulated. No change in mRNA level of hypoxia-inducible factor 1 and alpha subunit. Apoptosis in the lesions was obvious. Nuclear factor-kappa B and mitogen-activated protein kinase 1 mRNA levels were up-regulated. No differences were observed in all parameters with vitamin E treatment.	EGCG suppresses the development of experimental endometriosis through the anti-angiogenic mechanism.	[33]
1. 50 mg/kg/day EGCG 2. 20 mg/kg/day vitamin E 3. Saline via intraperitoneal injection	Daily for 3 weeks	1. Hetero-transplants of eutopic endometrium from patients with stage III endometriosis into severely compromised immunodeficient (SCID) ovariectomised mice 2. Mouse endometrial tissues transplanted into SCID mice	EGCG inhibited microvessel development in endometriotic implants. EGCG suppressed vascular endothelial growth factor C (VEGF-C) and tyrosine kinase receptor VEGF receptor 2 (VEGFR2) expression. EGCG down-regulated VEGFC/VEGFR2 signalling through c-JUN, interferon-γ, matrix metalloproteinase 9 and chemokine (C-X-C motif) ligand. No differences were observed in all parameters with vitamin E treatment.	EGCG inhibited angiogenesis and suppressed VEGFC/VEGFR2 expression and signalling pathway.	[39]
1. EGCG 50 mg/kg/day 2. 200 mL PBS (vehicle) via intraperitoneal injection	Daily for 14 days	1. Hetero-transplants of endometrium from patients with endometriosis into severely compromised immunodeficient (SCID) ovariectomised mice	Significant lower scores for both Sirius red and Masson trichrome staining in EGCG treated mice. Significant lower score human CD10 staining in untreated mice than in treated mice.	EGCG inhibits fibrosis in endometriosis	[41]
1. 20 mg/kg EGCG 2. 100 mg/ kg EGCG 3. Resveratrol 10 mg/kg 4. Resveratrol 25 mg/kg Via oral route	Daily for 4 weeks	Transplantation of mouse uterine horns to the mouse bowel mesentery to induce endometriotic-like lesions in a BALB/c mouse model	EGCG and resveratrol significantly reduced the number and volume of endometriotic lesions. EGCG and resveratrol significantly diminished cell proliferation, reduced vascular density and increased apoptosis within the lesions.	EGCG treatment inhibits the development and reduces the size of endometriotic lesions by reducing cell proliferation and increasing apoptotic activity.	[35]
1. 50 mg/kg pro-EGCG (EGCG octaacetate). 2. 50 mg/kg EGCG 3. 20 mg/kg Vitamin E via intraperitoneal injection	Daily for 4 weeks	Transplantation of endometrial tissues from transgenic luciferase expressing (CMV-Luc) mice into non-luminescent NOD-SCID mice	EGCG and pro-EGCG significantly decreased endometrial implant growth from the 2nd week to the 4th week. EGCG and pro-EGCG significantly reduced lesion size and weight, inhibited functional and structural microvessels in the lesions and enhanced apoptosis. Inhibition by pro-EGCG in all the angiogenesis parameters was significantly greater than that by EGCG. Pro-EGCG had better bioavailability and greater antioxidation and anti-angiogenesis capacities than EGCG. No differences were observed in all parameters with vitamin E treatment.	Pro-EGCG significantly inhibited the development, growth, and angiogenesis of experimental endometriosis in mice with greater efficacy, better bioavailability, and greater anti-oxidation and anti-angiogenesis capacities than EGCG.	[36]
1. 65 mg/kg EGCG 2. 200 mL DMSO (vehicle) intraperitoneally	Daily for 14 days	Transplantation of endometrium and ovarian follicles to Syrian golden hamster dorsal skinfold chamber model	EGCG group had significant reduction in the number and density of microvessels development within the endometriotic lesions but not in ovarian follicles. EGCG group decreased in centerline red blood cell velocity and volumetric blood flow within the endometriotic lesions but not in ovarian follicles. EGCG treatment induced the regression of endometriotic lesions.	EGCG prevents the establishment of new endometriotic lesions.	[37]
1. 8.333 mg/mL EGCG 2. 0.3 μg/μL decitabine 3. Saline Route: intraperitoneal	Once in 2 days for 16 days	Hetero-transplants of human endometrium into BALB/c female nude mice	Ectopic lesion growth: Increased from 4th to 16th day in the control group, Increased in the 0–8th day and decreased in the 8–16th day in the EGCG and decitabine group. Positive expression rate of E-cadherin: The decitabine group had a higher rate than the EGCG group, and the EGCG group had a higher rate than the control group. DNA methylation status of E-cadherin promoter region: The control group had a higher level than the EGCG group, and the EGCG group had a higher level than the decitabine group.	EGCG may inhibit the growth of the endometrial lesion, affect the expression of E-cadherin and reduce the status of DNA methylation of the E-cadherin promoter region.	[38]

**Table 3:** In vitro studies found related to the effects of green tea on endometriosis.

Treatment	Treatment Duration	Type of Cell	Findings	Conclusion	References
1. EGCG (10–50 µM) 2. Vitamin E (20 µM),	4 h	Human microvascular endothelial cells	EGCG suppressed VEGF-C expression and reduced VEGFR-2 and ERK activation.	EGCG inhibited angiogenesis and suppressed VEGFC/VEGFR2 expression and signalling pathway	[39]
1. EGCG 50 or 100 µM 2. N-acetyl-L-cysteine 5 or 100 µM	8–24 h	Endometrial and endometriotic stromal cells	EGCG significantly inhibited cell proliferation, migration and invasion. EGCG significantly decreased the TGF-β1-dependent increase in the mRNA expression of αSMA, Col-I, CTGF and FN mRNAs (fibrotic marker). EGCG significantly attenuated the cell-mediated contraction of collagen gels at 8, 12 and 24 h. EGCG inhibited TGF-β1-stimulated activation of MAPK and Smad signalling pathways.	EGCG inhibits fibrosis in endometriosis	[41]
1. EGCG (0, 20, 40, 80 and 100 µM) 2. Resveratrol (0, 25, 50 and 100 µM)	24 h	Primary cultures of human endometrial epithelial cells from patient with endometriosis	Reduction in cell proliferation and increase in apoptosis.	EGCG treatment inhibits the development of endometriotic lesions by reducing cell proliferation and increasing apoptotic activity.	[35]
1. 40 µM EGCG. 2. 1 µM 17β-estradiol 3. 40 µM EGCG + 1 µM 17β-estradiol		Isolated hamster endometrial stromal and glandular cells	EGCG suppressed E2-stimulated activation, proliferation and VEGF expression of endometrial cells.	EGCG prevents the establishment of new endometriotic lesions.	[37]

**Table 4:** Studies found related to the effects of green tea catechins on PCOS.

Treatment	Treatment Duration	Type of Study	Findings	Conclusion	References
1. Green tea tablet with unspecified dose 2. Placebo-capsules filled with wheat flour	Daily for 12 weeks	Randomized double-blind clinical trial on 60 overweight and obese PCOS Women aged between 20 to 40 years old	Significant reduction in weight and fasting insulin and testosterone levels in the green tea treated group compared with the placebo group.	The consumption of green tea by overweight and obese women suffering from PCOS leads to weight loss, a decrease in fasting insulin, and a decrease in the level of free testosterone.	[46]
1. Tablets equivalent to 500 mg green tea (C. Sinensis L.) leaf powder 2. Placebo-tablet corn starch	Daily for 45 days	Randomized double-blind placebo-controlled trial on 45 PCOS women aged 18–55 years with BMI range from 20–35 kg/m^2^	No change found in height, weight, BMI, WC, HC, waist-to-hip ratio or body fat between the treated and placebo groups. Significant reduction in weight, BMI, WC and body fat percentage after 45 days in the treated group. No significant difference in inflammatory marker TNF-α, IL-6 and hs-CRP levels. Significant positive correlations of nonsignificant decrease in the levels of IL-6 after reduction in body weight, BMI, waist circumference, hip circumference and body fat percentage was reported in the treated group.	Consumption of green tea tablets did not cause any effect on inflammation biomarkers in PCOS women. However, it may be effective as a complementary treatment for weight control in PCOS women.	[48]
1. Capsules containing 2% freeze-dried tea powder, equivalent to 540 mg epigallocatechin-3-gallate. 2. Placebo capsule-unspecified	Daily for three months	Randomized placebo-controlled trial on obese PCOS Women aged from 25–40 Years with BMI more than 28 kg/m^2^	No significant difference in body weight, BMI and body fat content between treatment and placebo group. No significant difference in testosterone, SHBG, free androgen, androstenedione, DHEA-S, FSH and LH levels between treatment and placebo group. No significant difference in fasting insulin, fasting glucose, 2 h post-load glucose, fasting G:I ratio, fasting leptin, fasting cholesterol, fasting HDL and LDL cholesterol levels between the treatment and placebo groups. Small but significant increase in triglyceride level in the treatment group after 3 months.	Green tea supplementation did not significantly reduce body weight in obese women with PCOS, nor did it alter the glucose or lipid metabolism.	[49]
1. 50,100 and 200 mg/kg body weight of hydro-alcoholic green tea extract 2. Saline	Intraperitoneally daily for 10 days	Adult female Wistar rats treated with estradiol valerate to induce PCOS	Significant decrease in the levels of LH and testosterone but no significant change in FSH level. Significant reduction in fasting glucose level. No changes in the level of insulin. Significant decrease in the HOMA-calculated insulin resistance. Significant decrease in body weight. Increase in the number of follicles and corpus luteum and reduced number of cystic follicles.	Green tea consumption causes modulating gonadotropin levels, reducing insulin resistance, losing rats’ weights and improving the ovarian morphology.	[47]

**Table 5:** Studies found related to the effects of green tea catechins on dysmenorrhea.

Treatment	Treatment Duration	Type of Study	Findings	Conclusion	References
Green tea intake	Not specified	A cross-sectional study involving reproductive age women in Shanghai, China.	Consumption of green tea was associated with a lower prevalence of dysmenorrhea. The relationship was stronger in moderate-to-severe dysmenorrhea than in mild dysmenorrhea.	Consumptions of green tea was associated with lower prevalence of dysmenorrhoea.	[57]
1. 5 mg/kg EGCG 2. 50 mg/kg EGCG intraperitoneally	Daily for 3 weeks	Mice treated with 1 mg/kg tamoxifen to induce adenomyosis	EGCG treatment: Suppressed myometrial infiltration. Improved generalised hyperalgesia. Reduced uterine contractility. Lowered plasma corticosterone levels	EGCG shows a benefit in treating adenomyosis in an animal study.	[58]

## Data Availability

The data presented in this study are available on request from the corresponding author.
